# Effect of periodic number of [Si/Sb_80_Te_20_]_*x*_ multilayer film on its laser-induced crystallization studied by coherent phonon spectroscopy

**DOI:** 10.1186/1556-276X-7-638

**Published:** 2012-11-22

**Authors:** Weiling Zhu, Changzhou Wang, Mingcheng Sun, Simian Li, Jiwei Zhai, Tianshu Lai

**Affiliations:** 1 Department of Physics, School of Science, Guangdong University of Petrochemical Technology, Maoming, Guangdong, 525000, China; 2 State-Key Laboratory of Optoelectronic Materials and Technologies, School of Physics and Engineering, Sun Yat-Sen University, Guangzhou, Guangdong, 510275, China; 3 Functional Material Research Laboratory, Tongji University, Shanghai, 200092, China; 4 Division of Energy and Environmental Research, Shanghai Advanced Research Institute, Chinese Academy of Sciences, Shanghai, 201210, China

**Keywords:** Crystallization, Coherent phonon spectroscopy, Optical phase change memory

## Abstract

The periodic number dependence of the femtosecond laser-induced crystallization threshold of [Si(5nm)/Sb_80_Te_20_(5nm)]_*x*_ nanocomposite multilayer films has been investigated by coherent phonon spectroscopy. Coherent optical phonon spectra show that femtosecond laser-irradiated crystallization threshold of the multilayer films relies obviously on the periodic number of the multilayer films and decreases with the increasing periodic number. The mechanism of the periodic number dependence is also studied. Possible mechanisms of reflectivity and thermal conductivity losses as well as the effect of the glass substrate are ruled out, while the remaining superlattice structure effect is ascribed to be responsible for the periodic number dependence. The sheet resistance of multilayer films versus a lattice temperature is measured and shows a similar periodic number dependence with one of the laser irradiation crystallization power threshold. In addition, the periodic number dependence of the crystallization temperature can be fitted well with an experiential formula obtained by considering coupling exchange interactions between adjacent layers in a superlattice. Those results provide us with the evidence to support our viewpoint. Our results show that the periodic number of multilayer films may become another controllable parameter in the design and parameter optimization of multilayer phase change films.

## Background

Sb-rich Sb-Te binary alloy is shown as a kind of high-speed growth-dominated phase change material [1,2] having fast crystallization rate, low archival stability, and high media noise [2,3]. It is also shown that Bi doping into Sb_80_Te_20_ can further reduce crystallization time but lead to a low crystallization temperature or poor archival stability [4]. In contrast, by adding Ge, Ag, In, and Ga into Sb_70_Te_30_ or Sb_80_Te_20_, a good archival stability is achieved, but the crystallization rate slows [1,2,5]. Therefore, it seems difficult to simultaneously optimize crystallization rate and temperature only by controlling composition. Recently, SbTe-based multilayer phase change films, such as GeTe/Sb_70_Te_30_[6], Si/Sb_80_Te_20_[7], and Ga_30_Sb_70_/Sb_80_ Te_20_[8], have been focused on because they provide more controllable degrees of freedom besides composition, such as the thickness of single layers, periodic number, etc. Yang et al. have shown the simultaneous optimization of high crystallization temperature and low storage power consumption of GeTe/Sb_70_Te_30_ multilayer films by adjusting the thickness ratio of GeTe and Sb_70_Te_30_ layers [6]. Wang et al. found that crystallization temperature and resistivity of the multilayer films Si/Sb_80_Te_20_ and Ga_30_Sb_70_/Sb_80_Te_20_ could be increased with the decrease of Sb_80_Te_20_ layer thickness or the increase of Si and Ga_30_Sb_70_ layer thickness [7,8]. However, the effect of another controllable parameter, periodic number or layer number of multilayer films, on the behavior of multilayer films has not been studied yet. It is possible to simultaneously optimize multiple parameters of multilayer films such as high crystallization rate, good thermal stability, and low power consumption by controlling layer thickness, periodic number, and composition.

In this article, the effect of the periodic number of Si (5 nm)/Sb_80_Te_20_ (5nm) multilayer films on laser-induced crystallization has been studied by coherent phonon spectroscopy. It is found that the degree of crystallization increases with irradiation power, and the power threshold of laser-induced crystallization of the multilayer films can be obviously reduced with the increase of the periodic number. Coherent optical phonon spectra reveal laser-induced crystallization processes. The mechanism of periodic number dependence is also discussed.

## Methods

### Sample

The samples studied consist of one, two, or ten periods of Si/Sb_80_Te_20_ films, where both the Sb_80_Te_20_ and Si layers are 5-nm thick and are grown on glass substrates by radio frequency magnetron sputtering using Sb_80_Te_20_ and Si targets. The Sb_80_Te_20_ layer is firstly deposited on the glass substrate, and then, the Si layer is deposited on top. This procedure is repeated appropriate times to obtain multilayer films with a desired periodic number. All depositions are performed at room temperature to ensure as-deposited films in an amorphous phase. The details on the conditions and procedures of the multilayer film preparation were described elsewhere [7].

### Experiment description

Time-resolved pump-probe photo-reflectivity spectroscopy is used to study the coherent phonon dynamics of the amorphous multilayer films. The details on the pump-probe experiment system have been described in previous reports [9]. A train of 60-fs-pulse laser from a self-mode-locked Ti:sapphire laser oscillator with a central wavelength of 840 nm and a repetition rate of 94 MHz was directed into a standard pump-probe setup. The emerging two parallel beams with a diameter of approximately 3 mm, a strong pump and a weak probe, are focused on the same area on the sample surface by a convex lens of 50 mm focal length. The focused spot is measured as about 35 μm in diameter. In experiments, the samples are first irradiated by a given pump power (referred to as irradiation power below) for a few seconds to induce phase change. Then, the pump power is reduced down to 15 mW, and the irradiated area is measured *in situ* by transient reflectivity change. Such measurement is repeated on a fresh spot, after which it is irradiated by a different irradiation power. All measurements are performed at room temperature and under a low pump power of 15 mW to avoid any pump-induced phase change during the measurement. It is noteworthy that laser power of 1 mW corresponds to an energy fluence of approximately 1.16 μJ/cm^2^ or a peak power density of approximately 19.3 MW/cm^2^.The resistance changes of the related samples versus temperature are measured as described in reference [7].

## Results and discussion

### *In situ* characterization of laser irradiation crystallization

**Figure 1:**
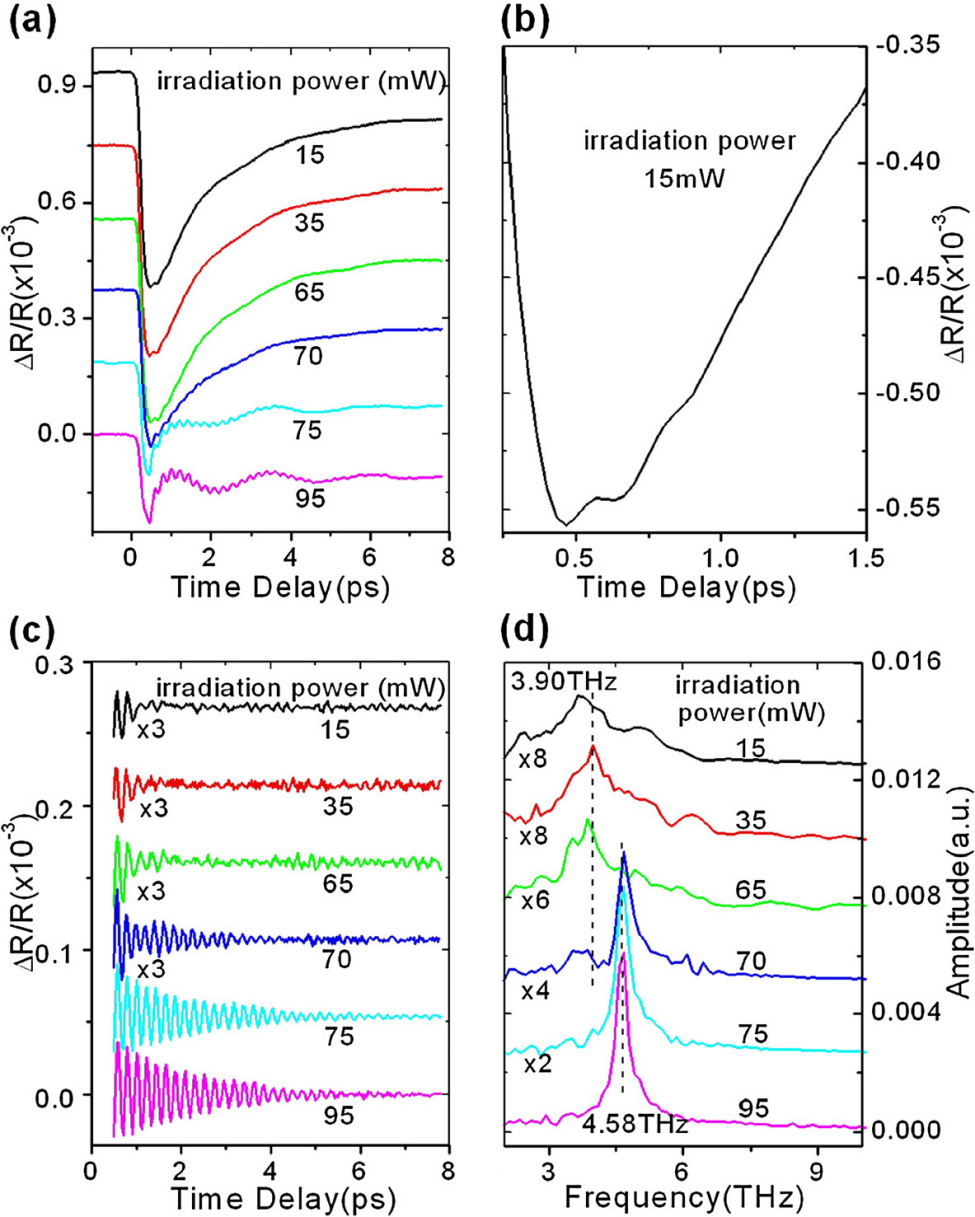
**Transient photo-reflectance changes and corresponding FFT spectra.(a)** Transient photo-reflectance changes taken on the amorphous [Si/Sb_80_Te_20_]_2_film at different laser irradiation powers but the same pump power of 15 mW during the measurement. **(b)** Amplified plot with 15-mW irradiation power. **(c)** Transient oscillation of COPs retrieved from **(a)**. **(d)** FFT spectra corresponding to **(c)**.

Transient photo-reflectance changes are first taken on a two-period [Si/Sb_80_Te_20__2_ amorphous film and plotted in Figure 1a with an increasing laser irradiation power from 15 to 95 mW but the same pump power of 15 mW during measurements. Figure 1b shows an amplified plot with irradiation power of 15 mW. It is seen that transient traces are changed obviously with increasing irradiation power. It is worth reminding that each transient trace is taken under the same low pump of 15 mW. Thus, the change of transient traces reflects the phase change induced by different laser irradiation powers. It is evident that an oscillatory component is superimposed on a normal carrier dynamic profile, which is so-called coherent phonon spectroscopy (CPS) [9-13], and reflects the vibration of coherent optical phonons (COPs) excited by femtosecond pump pulses. The oscillatory and non-oscillatory components are separated by digital low-pass filtering [12] on the transient data in Figure 1a. The oscillating components are plotted in Figure 1c. They are fast Fourier-transformed (FFT). Corresponding FFT spectra are plotted in Figure 1d and show that an irradiation power threshold existed for laser-induced crystallization. Below 70 mW, FFT spectra show a peak at 3.90 THz, whereas up to 70 mW and above, a new peak appears at approximately 4.58 THz, which implies the occurrence of laser-induced phase change because different frequency COP modes correspond to different microstructures. It was already shown by Raman scattering that the peak at 3.90 THz arises from characteristic phonon mode of amorphous Sb_80_Te_20_ films [9], while the new peak at 4.58 THz may be assigned to A_1g_ mode of crystalline Sb [14,15], implying that the excess Sb in the Sb_80_Te_20_ layer is crystallized during laser irradiation above 70 mW.

In addition, a slow oscillation with a period of about 2 ps is observable in Figure 1a. It should originate from the vibration of the folded acoustic phonon generated by the periodic structure of the sample. Such folded acoustic phonon has been described in details in reference [9] and is no longer discussed here.

**Figure 2:**
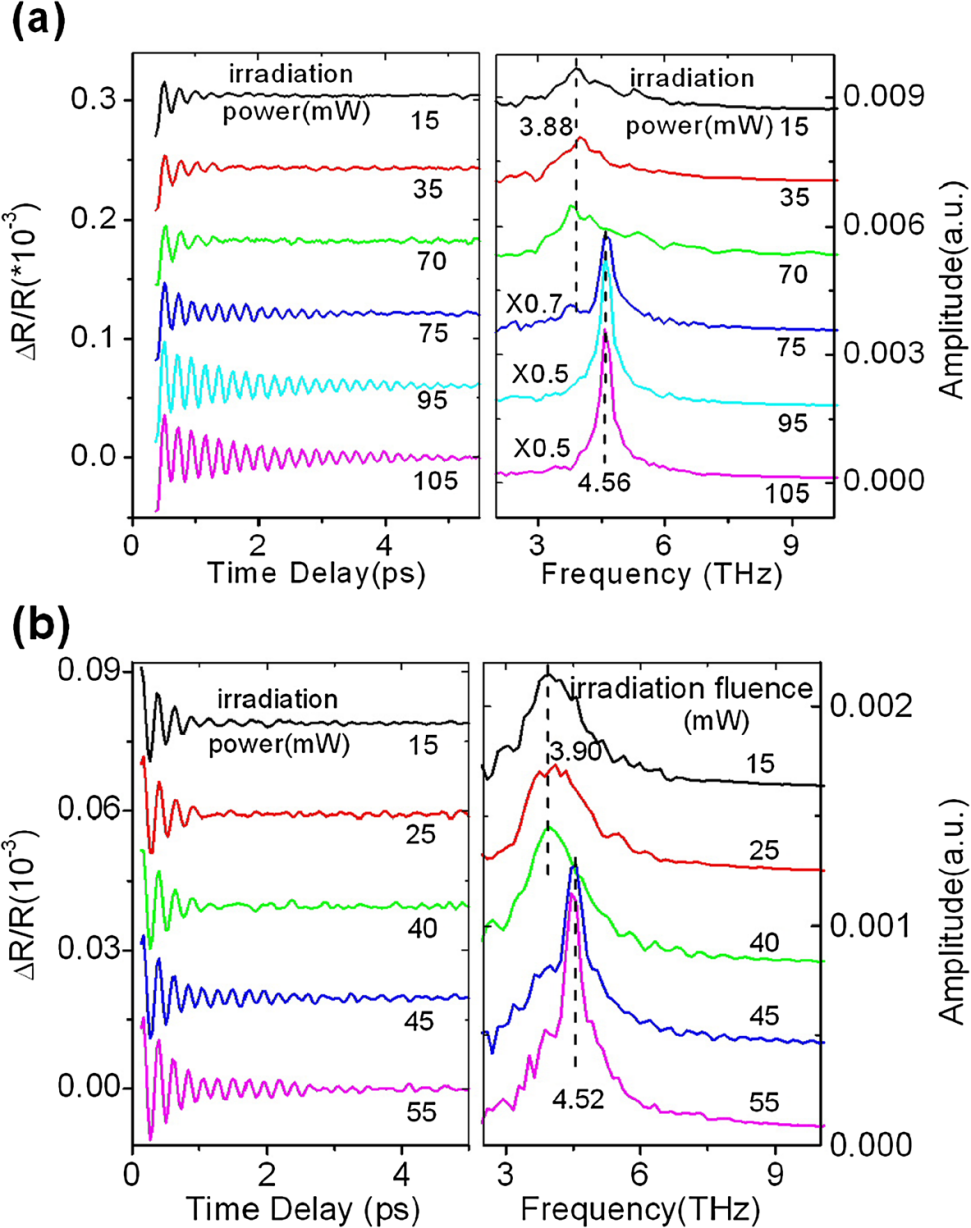
**Transient oscillation of COPs (left) and the corresponding FFT spectra (right).(a)** [Si/Sb_80_Te_20_]_1_ film and **(b)** [Si/Sb_80_Te_20_]_10_ film under different laser irradiation powers.

To study the effect of the periodic number of multilayer films on laser-induced crystallization, two other samples, one period and ten periods of Si/Sb_80_Te_20_ films, are measured by the same method mentioned before. Their transient oscillation components are plotted in the left column of Figure 2a,b. Their FFT spectra are also obtained and plotted in the right column, respectively. It is obvious that laser-induced crystallization has taken place with increasing irradiation power because new peaks at approximately 4.55 THz occur in the FFT spectra at above some irradiation power, but different crystallization threshold power occurs for one and ten periods of Si/Sb_80_Te_20_ films. One can see from Figures 1 and 2 that one, two, and ten periods of Si/Sb_80_Te_20_ films have different laser irradiation crystallization thresholds of 75, 70, and 45 mW, respectively, revealing an obvious periodic number dependence of the laser irradiation crystallization threshold. Therefore, the periodic number of multilayer films may become an effective degree of freedom to control the crystallization threshold of multilayer films.

### Mechanism of periodic number dependence of laser-induced crystallization threshold of multilayer films

To understand the mechanism of the periodic number dependence of the crystallization power threshold, we calculate the reflectivity of the three amorphous periodic film samples studied before according to the multiple-beam interference theory of multilayer film [16]. The results show that the reflectivities of the three samples are 20.67%, 31.03%, and 32.73%, respectively, for one, two, and ten periods of Si/Sb_80_Te_20_ films. Ten periods of Si/Sb_80_Te_20_ film has the highest reflective loss, but the lowest crystallization threshold. Consequently, the periodic number dependence of the crystallization power threshold cannot be explained by a reflective loss.

**Figure 3:**
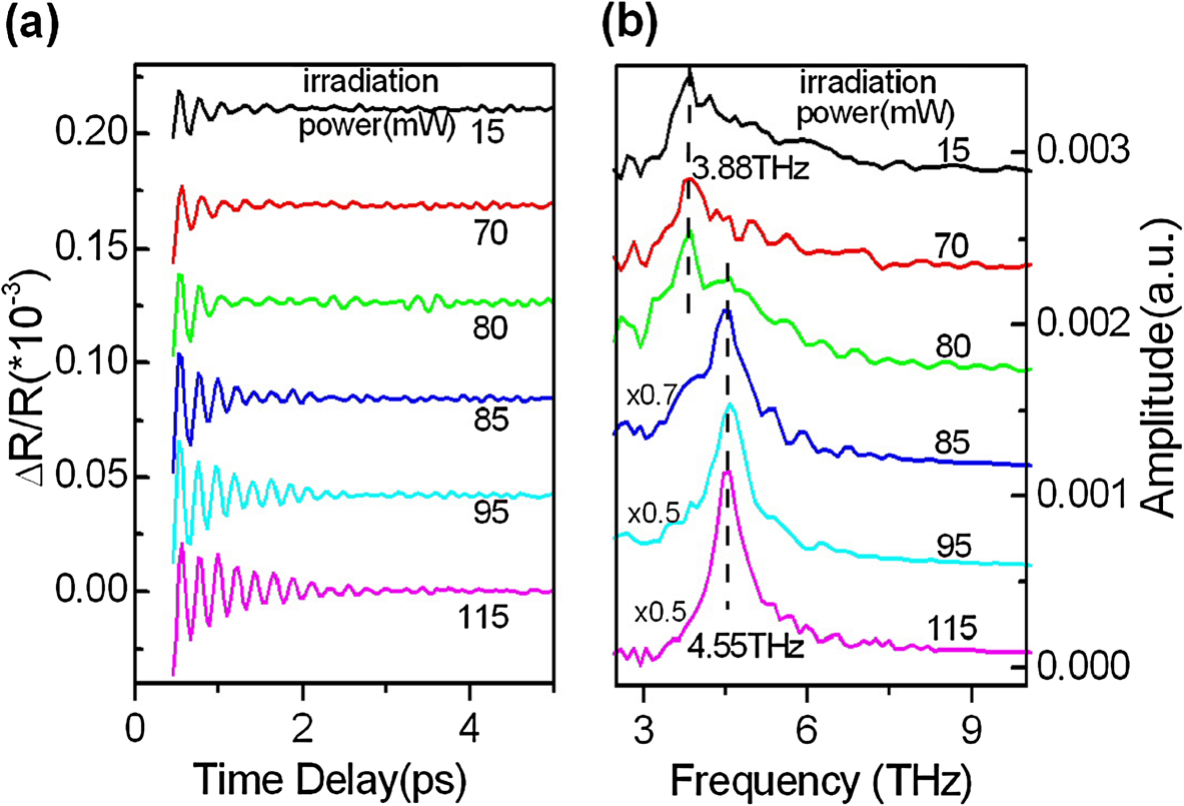
**Transient COP oscillation of a single layer of 5-nm-thick Sb**_**80**_**Te**_**20**_**film (a).(b)** FFT spectra corresponding to **(a)**.

On the other hand, heat conductivity effect may influence the laser crystallizing threshold. To test this possibility, a single layer of 5-nm-thick Sb_80_Te_20_ film is measured in the same way as described in the ‘Methods’ section. The transient oscillation components are plotted in Figure 3a. Their FFT spectra are plotted in Figure 3b and show that a new peak appeared at approximately 4.55 THz when the irradiation power increases up to 85 mW, implying a crystallization threshold of 85 mW for the single-layer Sb_80_Te_20_ film. The value of 85 mW is larger than the threshold of 75 mW for a single period of Si/Sb_80_Te_20_ film, which does not seem to support the possible mechanism that heat conductivity dominates the crystallization threshold of multilayer Si/Sb_80_Te_20_ films because Si layer has better heat conductivity and hence better heat exchange with Sb_80_Te_20_ layer than air. In this way, a single period of Si/Sb_80_Te_20_ film would have a higher crystallization threshold than single-layer Sb_80_Te_20_ film. However, the fact is opposite.

In addition, the glass substrate effect needs to be considered because the substrate has different influences on the three samples due to the limited penetration depth of the laser. According to the extinction coefficients of amorphous silicon (*k* ≈ 0 [17]) and amorphous Sb_80_Te_20_ (*k* ≈ 2.0 [1]) at a wavelength of 840 nm, the penetration depth of laser in the sample can be estimated as being approximately 33nm. As a result, the single- and double-period samples are penetrated by the laser, but the ten-period sample almost cannot be penetrated. Consequently, the substrate may have different influences on crystallization, such as possible obvious different effects on the crystallized zone in heat sink. To check for possible substrate effect, similar experiments to the ones described before are repeated on three samples, but pumping and probing are made from the substrate side, that is, the light goes through the substrate and interacts with the film. In such way, the substrate should have the same effect on the crystallized zone. However, three different crystallization thresholds of 50, 45, and 35 mW are still found for single-, double-, and ten-period films, respectively, which also decreased with increasing periodic number. This variation tendency with periodic number still cannot be explained by reflective losses because the reflectivity of the sample being incident from the substrate side is still increased with increasing periodic number, that is, 10.62%, 16.89%, and 23.13%, respectively, for single-, double-, and ten-period samples. Therefore, the effect of the substrate on crystallization can be ruled out. As for the fact that the crystallization threshold of laser incident from the substrate side becomes smaller than one incident from the film side, it can be explained well by the fact that the reflective loss of laser incident from the substrate side is smaller than one from the film side. Actually, one can find that the real absorbed power threshold of each sample is almost identical regardless of laser incidence from the substrate or film side if one calculates the absorptivity of each sample based on multiple-beam interference of multiple layer films for the two incidences of each sample from the substrate and film sides.

The remaining possible mechanism may be heat exchange coupling in a superlattice. It is possible because the layer thickness is only 5 nm in our multilayer films. There may be a strong exchange interaction between the Si layer and the Sb_80_Te_20_ layer. Therefore, the periodic multilayer films actually form a superlattice, a new type of material [9]. In a superlattice, the coupling effect between adjacent layers may lead to the parameters of superlattices different from those of elementary materials composing the superlattice. Actually, a previous report on crystallization temperature change of multilayer films with layer thickness has indirectly shown such evidence [7]. In other words, the structure determines the properties of superlattices. Rama et al. also showed that the coupling effect between adjacent layers makes the periodic film equivalent to an alloy layer [18]. Wang et al. [7,19] gave out an experiential formula, $T_{C}=T_{\mathrm{ac}}\left(1+A_{1}e^{-\frac{d}{4l_{0}}}\right)$, to describe the dependence of crystallization temperature of the alloy layer on its thickness or periodic number of multilayer films, where *T*_c_ represents the crystallization temperature of the periodic film, *d* is the total thickness of the periodic films, *T*_ac_ represents the crystallization temperature of a very thick periodic film (d→∞), *A*_1_ is a complex parameter related to interface free energies, and *l*_0_ is the average screening or bonding length associated with the range of interatomic forces.

From the formula above, the crystallization temperature of multilayer films is reduced with the increase of its periodic number [7,19]. In other words, the crystallization power threshold of multilayer films is decreased when its periodic number is increased. It agrees very well with our experimental results shown in following section. Therefore, the dependence of laser-irradiated crystallization threshold on the period number of multilayer films may be ascribed to the superlattice effect of multilayer films.

**Figure 4:**
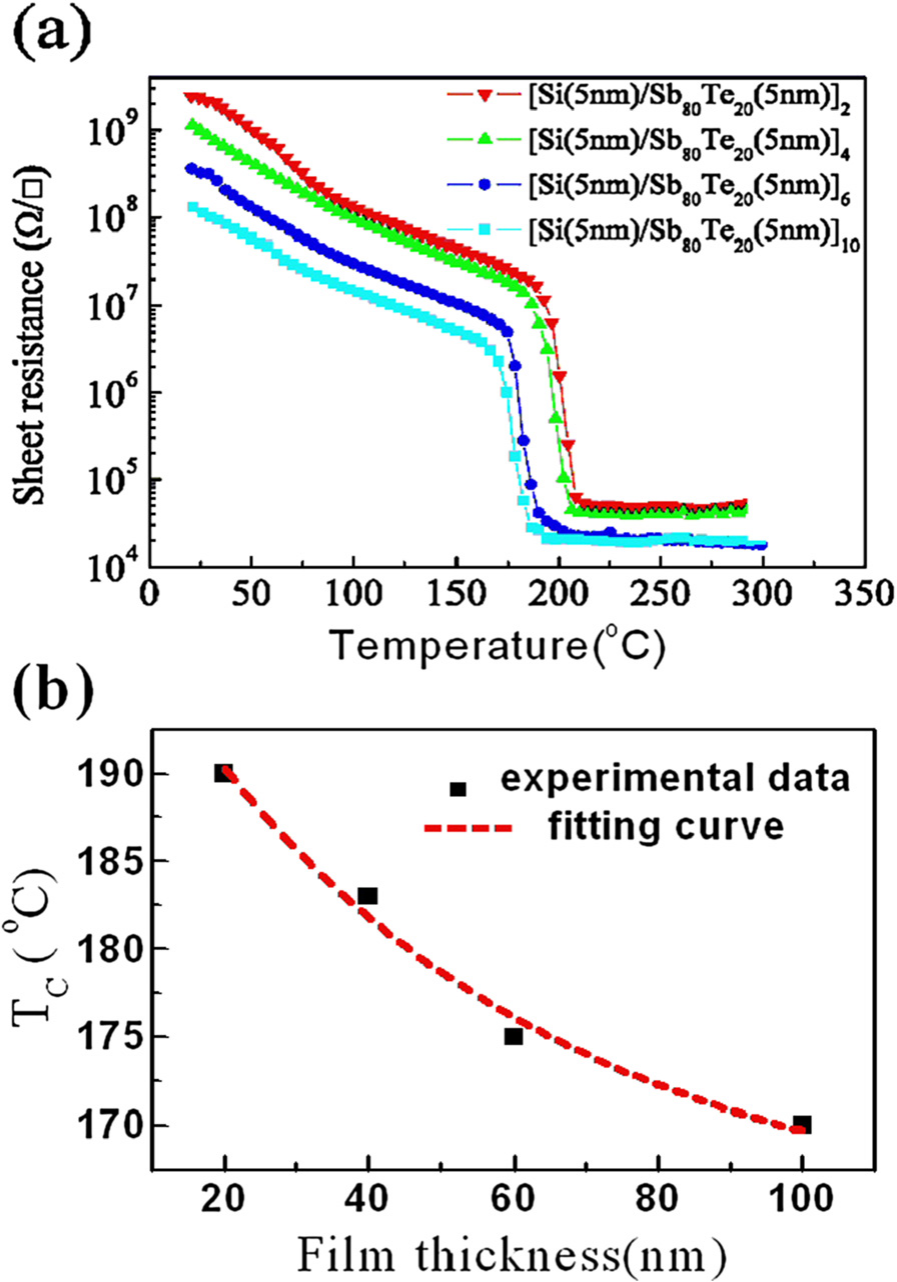
**Temperature dependence of sheet resistance of [Si/Sb_80_Te_20_]**_***x***_**films. (a)**. The crystallization temperature decreases with the increase of the periodic number. **(b)** Crystallization temperature as an exponential function of the total thickness of [Si/Sb_80_Te_20_]_*x*_ films.

To further test the effect quantitatively, we measure the crystallization temperature of four samples, [Si/Sb_80_Te_20__*x*_ films with *x* = 2, 4, 6, and 10, by measuring the temperature dependence of sheet resistance, as described in reference [7]. Figure 4a shows the resistance-temperature curves of the four samples and exhibits four crystallization temperatures at 190°C, 185°C, 175°C, and 170°C for *x* = 2, 4, 6, and 10, respectively. The best fitting to the four crystallization temperatures with the formula above is shown in Figure 4b by a dashed line, which agrees very well with the filled-square experimental data points. Meanwhile, the best fitting also gives out parameters, *T*_ac_ = 164.3°C, *A*_1_ = 0.2348, and *l*_0_ = 12.7 nm. *T*_ac_ = 164.3°C means that the crystallization temperature of the structure of the Si(5nm)/Sb_80_Te_20_(5nm) films will lower down to 164.3°C as its period number approaches infinity. The excellent fitting to the experimental crystallization temperature data with the experiential formula again supports our viewpoint above. Furthermore, the parameter of bonding length, *l*_0_ = 12.7 nm, in the empirical formula is obviously larger than the layer thickness of 5 nm, which also suggests a strong interatomic interaction between adjacent layers.

## Conclusions

The periodic number dependence of the femtosecond laser-induced crystallization of [Si/Sb_80_Te_20_]_*x*_ nanocomposite multilayer films has been characterized by CPS. The frequency of COP modes before and after crystallization appears at approximately 3.90 and 4.55 THz, respectively. The former is assigned to the phonon mode of amorphous Sb_80_Te_20_ layer, while the latter is assigned to the *A*_1g_ optical phonon mode of crystalline Sb and is not related to the periodic number. In addition, the crystallization power threshold decreases with the increase of the periodic number. The periodic number dependence of the power threshold is attributed to the crystallization temperature decrease with the increase of the periodic number and is considered originating from superlattice structure effects. The periodic number of the multilayer films provides another additional degree of freedom to simultaneously optimize multiple parameters of the multilayer phase change films.

## Abbreviations

FFT: Fast Fourier-transformed; COP: Coherent optical phonons; CPS: Coherent phonon spectroscopy.

## Competing interests

The authors declare that they have no competing interests.

## Authors’ contributions

CW, MS, and JZ carried out the sample preparation and electrical characterizations. WZ,SL, and TL conceived of the laser-induced crystallization studies and coordinated the experiment. All of the authors participated in the analysis of the data. WZ and TL wrote the manuscript. All authors read and approved the final manuscript.
